# Estimated pulse wave velocity predicts outcomes after acute ischemic stroke: a multinational cohort analysis

**DOI:** 10.1093/ajh/hpag066

**Published:** 2026-06-25

**Authors:** Nikolaos Kakaletsis, Naohisa Hosomi, Tomohisa Nezu, Patrik Michel, Thevoz Guillaume, Davide Strambo, Young Seo Kim, Wonjae Sung, Konstantinos Vemmos, Eleni Korompoki, Maurizio Acampa, Jukka Putaala, Lauri Tulkki, Matthias Hermann, Protazy Rejmer, Philip M Bath, Lisa J Woodhouse, Vasilios Kotsis, Elpida Athanasopoulou, Athanase D Protogerou, Haralampos Milionis, George Ntaios, Christos Savopoulos

**Affiliations:** First Propedeutic Department of Internal Medicine, Medical School, Aristotle University of Thessaloniki, AHEPA University Hospital, Thessaloniki, Greece; Department of Clinical Neuroscience and Therapeutics, Hiroshima University Graduate School of Biomedical Sciences, Hiroshima, Japan; Department of Clinical Neuroscience and Therapeutics, Hiroshima University Graduate School of Biomedical Sciences, Hiroshima, Japan; Stroke Center and Neurology Service, Department of Clinical Neurosciences, Centre Hospitalier Universitaire Vaudois and University of Lausanne, Lausanne, Switzerland; Stroke Center and Neurology Service, Department of Clinical Neurosciences, Centre Hospitalier Universitaire Vaudois and University of Lausanne, Lausanne, Switzerland; Stroke Center and Neurology Service, Department of Clinical Neurosciences, Centre Hospitalier Universitaire Vaudois and University of Lausanne, Lausanne, Switzerland; Department of Neurology, College of Medicine, Hanyang University, Seoul, Republic of Korea; Department of Neurology, College of Medicine, Hanyang University, Seoul, Republic of Korea; Department of Clinical Therapeutics of Alexandra Hospital, National and Kapodistrian University of Athens, Athens, Greece; Department of Clinical Therapeutics of Alexandra Hospital, National and Kapodistrian University of Athens, Athens, Greece; Stroke Unit, Department of Medical Sciences, Surgery and Neurosciences, “Santa Maria Alle Scotte” General Hospital, University of Siena, Siena, Italy; Department of Neurology, Helsinki University Hospital and University of Helsinki, Helsinki, Finland; Department of Neurology, Helsinki University Hospital and University of Helsinki, Helsinki, Finland; University Heart Center Zurich, Department of Cardiology, University Hospital Zurich, Zurich, Switzerland; University Heart Center Zurich, Department of Cardiology, University Hospital Zurich, Zurich, Switzerland; Stroke Trials Unit, Mental Health & Clinical Neuroscience, University of Nottingham, Nottingham, United Kingdom; Stroke Trials Unit, Mental Health & Clinical Neuroscience, University of Nottingham, Nottingham, United Kingdom; Third Department of Internal Medicine, Medical School, Aristotle University of Thessaloniki, Papageorgiou Hospital, Thessaloniki, Greece; Cardiovascular Prevention & Research Unit, Clinic & Laboratory of Pathophysiology, Department of Medicine, School of Health Sciences, National and Kapodistrian University of Athens, Athens, Greece; Cardiovascular Prevention & Research Unit, Clinic & Laboratory of Pathophysiology, Department of Medicine, School of Health Sciences, National and Kapodistrian University of Athens, Athens, Greece; Department of Internal Medicine, Medical School, University of Ioannina, University Hospital of Ioannina, Ioannina, Greece; First Propedeutic Department of Internal Medicine, Medical School, Aristotle University of Thessaloniki, AHEPA University Hospital, Thessaloniki, Greece; First Propedeutic Department of Internal Medicine, Medical School, Aristotle University of Thessaloniki, AHEPA University Hospital, Thessaloniki, Greece

**Keywords:** acute ischemic stroke, estimated pulse wave velocity, vascular aging, arterial stiffness, 24-hour blood pressure monitoring, prognosis, functional outcome

## Abstract

**Background:**

Estimated pulse wave velocity (ePWV), an easy to calculate proxy of carotid to femoral PWV (cfPWV), can be derived from 24-hour blood pressure monitoring (24h-BPM). Its role in acute ischemic stroke (AIS) has not previously been investigated in large multinational datasets.

**Methods:**

A pooled individual patient data analysis from 13 cohorts across seven countries was conducted, including 2933 AIS patients who underwent 24h-BPM during the hyperacute to subacute phase. ePWV was calculated using a validated equation. Associations with clinical characteristics, stroke severity, etiologic subtypes, and outcomes were assessed with logistic regression. The primary outcome was poor functional outcome at 90 days, defined as a modified Rankin Scale (mRS) >2.

**Results:**

Patients with higher ePWV presented with more severe strokes (median NIHSS 7 vs. 3, *P* < .001) and had poorer outcomes both at 90 days (63.7% vs. 23.7%, *P* < .001) and discharge (61.9% vs 29.1%, *P* < .001). ePWV was associated with poor outcome at 90 days (aOR: 1.24, 95% CI: 1.05-1.45) and discharge (aOR: 1.26, 95% CI: 1.12-1.42), per 1 m/s increase in ePWV, after adjustment for conventional risk factors and stroke severity. In patients ≤50 years, ePWV was highest in those with small vessel disease (SVD), who also had more cardiovascular risk factors than other stroke subtypes.

**Conclusions:**

ePWV derived from 24h-BPM is a simple, widely applicable measure of vascular aging that is associated with functional outcome after AIS. The higher ePWV observed in young patients with SVD underscores a potential early arteriosclerotic burden in this subgroup.

## Graphical abstract

**Graphical Abstract:**
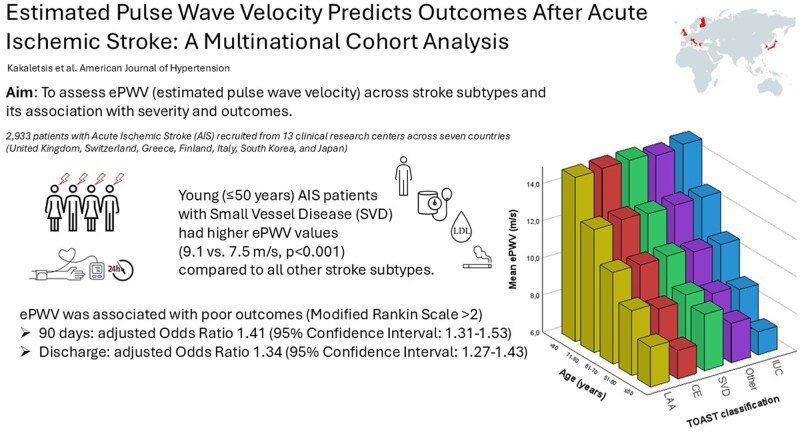

## Introduction

Despite advances in acute treatment and secondary prevention, patients with acute ischemic stroke (AIS) remain at high risk of poor functional outcomes, recurrent stroke, cardiovascular events, and mortality.^1–3^ Among important determinants of this residual risk, vascular aging, particularly arterial stiffening, has gained attention as a key contributor to cerebrovascular disease and a potential therapeutic target.^4–8^

Early vascular aging (EVA), characterized by premature arteriosclerosis and increased arterial stiffness, has been linked to higher rates of functional disability, recurrent events, and mortality after AIS.^9^^,^^10^ Pulse wave velocity (PWV), the gold standard for regional arterial stiffness assessment, is a strong predictor of cardiovascular outcomes^5^^,^^6^^,^^11^; however, its use in acute stroke settings may be limited by feasibility and availability constraints.^12^

Understanding the role of vascular aging in AIS may improve risk stratification and guide targeted therapeutic strategies. Estimated PWV (ePWV), derived from oscillometric blood pressure measurements, offers a feasible alternative and has been validated as a proxy of carotid to femoral PWV (cfPWV) in various populations.^13^ However, evidence on ePWV in AIS, particularly across stroke subtypes, severities, and its prognostic value, remains limited.

This study aimed to evaluate ePWV levels among different AIS etiologic subtypes and further investigate its associations with stroke severity, patient characteristics, and functional outcome.

## Methods

### Study population

This work is based on an individual patient data (IPD) pooled analysis that combined information from previously conducted interventional trials, observational studies, and prospective registries. The design, protocol, and eligibility criteria of these contributing studies have been reported in detail elsewhere.^14^^,^^15^ In summary, all studies enrolled adults (≥18 years) with AIS who underwent 24-hour blood pressure monitoring (24h-BPM) during the hyperacute, acute, or subacute phase after stroke onset. A comprehensive overview of the included cohorts, covering country of origin, design, recruitment period, sample size, mean age, sex distribution, median baseline stroke severity, stroke subtype, timing of 24h-BPM, and reported outcomes, is presented in Tables S1 and S2.

### Clinical and laboratory data

Baseline demographic and clinical characteristics collected were age, sex, body mass index (BMI), vascular risk factors (hypertension, diabetes mellitus, dyslipidemia), atrial fibrillation, coronary artery disease (CAD), previous stroke or transient ischemic attack (TIA), and smoking history. Patients were stratified into age groups by decade: ≤50, 51-60, 61-70, 71-80, and >80 years. From 24h-BPM, mean systolic blood pressure (SBP), diastolic blood pressure (DBP), and heart rate (HR) were derived. Estimated glomerular filtration rate (eGFR) was calculated using the CKD-EPI 2021 equation, and lipid profile measurements included total cholesterol, HDL cholesterol, LDL cholesterol, and triglycerides.

Stroke severity at admission was evaluated with the National Institutes of Health Stroke Scale (NIHSS).^16^ For analytic purposes, NIHSS values were grouped as follows: 0-4 (minor), 5-15 (moderate), 16-20 (moderate-to-severe), and 21-42 (severe).^17^ Where available, pre-stroke disability was assessed by the modified Rankin Scale (mRS) and dichotomized into independent (mRS ≤2) or dependent (mRS >2). Stroke etiology was classified using the Trial of Org 10172 in Acute Stroke Treatment (TOAST) criteria into large artery atherosclerosis (LAA), cardioembolic (CE), small-vessel disease (SVD), other determined causes, and infarct of undetermined cause (IUC).^18^ Data on acute management, including intravenous thrombolysis and mechanical thrombectomy, were also documented.

Ethical approval for the pooled analysis was obtained from the Institutional Review Board of the Aristotle University of Thessaloniki (approval number 1270/29-10-2020). All original studies complied with their local institutional ethics requirements.

### Estimated pulse wave velocity

Estimated PWV has been proposed as a proxy of the reference cfPWV and has been shown to predict cardiovascular outcomes independently of conventional risk factors.^13^ Its prognostic value extends to drug response and to long-term all-cause and cardiovascular mortality.^19^ ePWV was calculated using the validated equation by Greve et al,^20^ derived from the Reference Values for Arterial Stiffness Collaboration,^21^ specifically for individuals with cardiovascular risk factors. The formula is as follows^13^: ePWV = 9.58748315543126 − 0.402467539733184 * age + 4.56020798207263 * 10^−3^ * age^2^ − 2.6207705511664 * 10^−5^ * age^2^ * MAP + 3.1762450559276 * 10^−3^ * age * MAP − 1.83215068503821 * 10^−2^ * MAP. Mean arterial pressure (MAP) was derived from 24h-BPM values using the following formula: MAP = (24h-DBP) + 0.4*(24h-SBP − 24h-DBP).

### Outcomes

Functional outcome was assessed using the modified Rankin Scale (mRS), with the primary outcome measured at 90 days and the secondary outcome at discharge. Poor outcome was defined as mRS >2.^22^

### Statistical analysis

Continuous variables were summarized as mean ± standard deviation, except for NIHSS, which was expressed as median with interquartile range (IQR). Categorical data were reported as frequencies and percentages. Normality was tested with the Kolmogorov-Smirnov test. Since none of the variables followed a normal distribution, comparisons between two groups were performed with the Mann-Whitney *U* test, while the Kruskal-Wallis test was used for multiple groups. Categorical variables were compared using Pearson’s chi-square or Fisher’s exact test, as appropriate.

Patients were stratified according to the median of the ePWV distribution, and comparisons of clinical characteristics, laboratory parameters, blood pressure, and outcomes were performed between these groups. Furthermore, mean ePWV values were compared between subgroups defined by stroke etiology (TOAST) and baseline stroke severity (NIHSS category).

Binary logistic regression was employed to assess the predictive value of ePWV for dichotomous outcomes (poor functional outcome at discharge and at 90 days). ePWV was modeled as a continuous variable, and effect estimates are expressed per 1 m/s increase. Multivariable models included variables with *P* < .10 in univariate analyses and with <10% missing data,^14^ excluding those used in the ePWV formula (24h-SBP, 24h-DBP) to avoid multicollinearity. Sensitivity analyses were performed to assess the robustness of the association between ePWV and outcomes after adjusting for age and 24h-SBP, 24h-DBP, and 24-hour pulse pressure (24h-PP). Additional models evaluated 24h-PP as an alternative marker of arterial stiffness. Multicollinearity among predictors was assessed using variance inflation factors (VIF) derived from linear regression models, with VIF >10 indicating severe collinearity. To further address shared variance between ePWV and its constituent variables, residualized ePWV variables were derived by regressing ePWV on age and 24h-PP. The resulting residuals were included in multivariable models together with age and 24h-PP. Patients with missing information on outcomes or covariates were excluded from the corresponding analyses.

All statistical tests were two-sided, with significance set at *P* < .05. Analyses were performed using IBM SPSS Statistics for Windows, version 29.0 (IBM Corp., Armonk, NY, 2022).

## Results

### General characteristics

This pooled analysis included 2933 patients with AIS recruited from 13 clinical research centers across seven countries (United Kingdom, Switzerland, Greece, Finland, Italy, South Korea, and Japan). The mean age was 72.0 ± 14.5 years, and 56.5% were male. Regarding vascular risk factors, 69.1% had hypertension, 43.8% dyslipidemia, 27.6% diabetes mellitus, 25.6% were current smokers, 24.6% had atrial fibrillation, and 22.6% had coronary artery disease (Table 1).

**Table 1:** Clinical and laboratory features of AIS patients by median ePWV (high vs low).

Variable	Valid values	All AIS patients	High ePWV (≥12.4 m/s)	Low ePWV (<12.4 m/s)
**ePWV (m/s)**	2933	12.2 ± 2.7	14.4 ± 1.3	10.1 ± 1.7
**Demographic/somatometric data**				
**Age (years)**	2933	72.0 ± 14.5	82.6 ± 6.3	61.4 ± 12.4
**Sex (male)**	2933	1658 (56.5%)	684 (46.6%)	974 (66.4%)
**BMI (kg/m^2^)**	2883	25.1 ± 4.5	24.6 ± 4.4	25.6 ± 4.5
**Clinical risk factors**				
**Hypertension**	2930	2024 (69.1%)	1170 (79.9%)	854 (58.3%)
**Diabetes**	2930	810 (27.6%)	405 (27.7%)	405 (27.6%)
**Dyslipidemia**	2929	1282 (43.8%)	639 (43.7%)	643 (43.9%)
**Atrial fibrillation**	2848	701 (24.6%)	490 (34.7%)	211 (14.7%)
**CAD**	2765	626 (22.6%)	402 (29.5%)	224 (16.0%)
**Prior stroke/TIA**	2795	517 (18.5%)	314 (21.4%)	203 (15.3%)
**Smoking**	2818	720 (25.6%)	212 (14.9%)	508 (36.4%)
**24h-BPM data**				
**24-h SBP (mmHg)**	2933	136.0 ± 20.2	141.8 ± 20.1	130.2 ± 17.8
**24-h DBP (mmHg)**	2933	78.7 ± 11.6	79.9 ± 12.2	77.5 ± 10.9
**24-h HR (bpm)**	2932	74.9 ± 13.8	72.5 ± 12.9	77.3 ± 14.3
**Laboratory data**				
**TCL (mmol/l)**	2613	4.8 ± 1.3	4.7 ± 1.1	5.0 ± 1.4
**TRG (mmol/l)**	2328	1.4 ± 1.0	1.3 ± 0.8	1.6 ± 1.2
**LDL (mmol/l)**	2553	2.9 ± 1.1	2.8 ± 1.0	3.0 ± 1.1
**HDL (mmol/l)**	2313	1.2 ± 0.4	1.3 ± 0.4	1.2 ± 0.4
**eGFR**	2774	72.6 ± 24.9	63.6 ± 22.1	81.7 ± 24.3
**Stroke data**				
**Pre-stroke mRS > 2**	2207	381 (17.3%)	312 (26.1%)	69 (6.8%)
**Admission NIHSS**	2738	5 (2-12)	7 (3-15)	3 (1-8)
***TOAST***				
**LAA**	2797	529 (18.9%)	250 (18.1%)	279 (19.7%)
**CE**		681 (24.3%)	449 (32.6%)	232 (16.4%)
**SVD**		455 (16.3%)	170 (12.3%)	285 (20.1%)
**Other**		274 (9.8%)	111 (8.0%)	163 (11.5%)
**IUC**		858 (30.7%)	399 (28.9%)	459 (32.4%)
**Acute interventions**				
**Thrombolysis**	2589	509 (19.7%)	222 (16.8%)	287 (22.7%)
**Thrombectomy**	737	50 (6.8%)	20 (4.9%)	30 (9.2%)
**Outcomes**				
**Discharge mRS > 2**	2307	1075 (46.6%)	761 (61.9%)	314 (29.1%)
**90 days mRS > 2**	1550	703 (45.4%)	535 (63.7%)	168 (23.7%)

Data are numbers (%) for categorical variables, mean ± SD for continuous variables, except NIHSS which is median (IQR).

Abbreviations: 24h-BPM: 24-hour blood pressure monitoring, AIS: acute ischemic stroke, BMI: body mass index, CAD: coronary artery disease CE: cardioembolic, DBP: diastolic blood pressure, eGFR: estimated glomerular filtration rate (ml/min/1.73m^2^), ePWV: estimated pulse wave velocity, HDL: high-density lipoprotein cholesterol, HR: heart rate, IUC: infarct of undetermined cause, LAA: large artery atherosclerosis, LDL: low-density lipoprotein cholesterol, NIHSS: National Institutes of Health stroke scale, Other: stroke of other determined causes, SBP: systolic blood pressure, SVD: small vessel disease or lacunar stroke, TCL: total cholesterol, TIA: transient ischemic attack, TOAST: Trial of Org 10172 in Acute Stroke Treatment, TRG: triglycerides.

Based on TOAST classification, 18.9% of patients were categorized as large artery atherosclerosis (LAA), 24.3% as cardioembolic (CE), 16.3% as small vessel disease (SVD), 9.8% as other determined etiologies, and 30.7% as infarcts of undetermined cause (IUC). The median baseline NIHSS score was 5 (IQR: 2-12). Overall, 19.7% of patients received intravenous thrombolysis. Functional outcome data were available for 2307 patients at discharge, of whom 46.6% had poor outcome (mRS >2). At 3 months, follow-up was available for 1550 patients, with 45.4% classified as having poor outcome (Table 1).

### Comparison between AIS patients with high ePWV versus low ePWV

The mean ePWV for the entire cohort was 12.2 ± 2.7 m/s, with a median value of 12.4 m/s. Patients with ePWV above the median were significantly older (82.6 vs 61.4 years, *P* < .001), more often female (53.4% vs 33.6%, *P* < .001), and had lower BMI (24.6 vs 25.6 kg/m^2^, *P* < .001). They exhibited higher prevalence of hypertension (79.9% vs 58.3%, *P* < .001), atrial fibrillation (34.7% vs 14.7%, *P* < .001), coronary artery disease (29.5% vs. 16.0%, *P* < .001), prior stroke/TIA (21.4% vs 15.3%, *P* < .001), and pre-stroke disability (26.1% vs 6.8%, *P* < .001). Conversely, smoking was less common among patients with higher ePWV (14.9% vs 36.4%, *P* < .001) (Table 1).

Stroke severity on admission was greater in the high ePWV group (median NIHSS 7 vs 3, *P* < .001). Despite presenting with more severe strokes, these patients were less likely to receive acute reperfusion therapy, both intravenous thrombolysis (16.8% vs. 22.7%) and thrombectomy (4.9% vs. 9.2%). Stratifying by stroke subtype, compared to patients with lower ePWV, those with higher ePWV had approximately double the proportion of cardioembolic strokes (32.6% vs. 16.4%) and nearly half the proportion of SVD (12.3% vs. 20.1%). Lipid levels were consistently lower in the high ePWV group compared with patients below the median ePWV (Table 1).

Outcomes were markedly worse in patients with elevated ePWV. Poor functional status (mRS >2) was more frequent both at discharge (61.9% vs 29.1%, *P* < .001) and at 3 months (63.7% vs 23.7%, *P* < .001) (Table 1).

### Comparison of ePWV across TOAST subtypes

Mean ePWV values differed among TOAST stroke subtypes. The lowest values were observed in patients with strokes of other determined causes or IUC (11.7 m/s), whereas cardioembolic strokes exhibited the highest mean ePWV (13.2 m/s) (Table 2).

**Table 2:** Comparison of mean ePWV in AIS patients by TOAST subtype.

TOAST (*n* = 2797)	LAA (*n* = 529)	CE (*n* = 681)	SVD (*n* = 455)	Other (*n* = 274)	IUC (*n* = 858)	*P*
**ePWV (m/s)**	12.3 ± 2.3	13.2 ± 2.4	11.8 ± 2.2	11.7 ± 2.6	11.7 ± 3.1	<.001*
***Age category (years)***						
**≤50**	8.2 ± 1.0 (29)	7.5 ± 0.8 (19)	9.0 ± 1.2 (41)	8.2 ± 1.2 (36)	7.2 ± 0.9 (175)	<.001*
**51-60**	9.5 ± 1.0 (54)	9.2 ± 1.4 (32)	9.5 ± 1.1 (66)	9.1 ± 1.1 (39)	8.9 ± 1.0 (68)	.018*
**61-70**	10.9 ± 1.1 (125)	10.9 ± 1.2 (97)	11.0 ± 1.0 (131)	10.8 ± 1.1 (58)	10.7 ± 1.1 (136)	.345
**71-80**	12.6 ± 1.1 (178)	12.7 ± 1.2 (215)	12.6 ± 1.0 (131)	12.7 ± 1.1 (69)	12.6 ± 1.1 (206)	.880
**>80**	14.8 ± 1.2 (143)	15.0 ± 1.4 (318)	14.9 ± 1.3 (86)	14.7 ± 1.2 (72)	15.0 ± 1.3 (273)	.625

Data are mean±SD (number), *P*value derived from the Kruskal-Wallis Test and statistically significant values (*P* < .05) have been indicated bold with an asterisk (*).

Abbreviations: AIS: acute ischemic stroke, CE: cardioembolic, ePWV: estimated pulse wave velocity, IUC: infarct of undetermined cause, LAA: large artery atherosclerotic, Other: stroke of other determined causes, SVD: small vessel disease or lacunar stroke, TOAST: Trial of Org 10172 in Acute Stroke Treatment.

**Figure 1:**
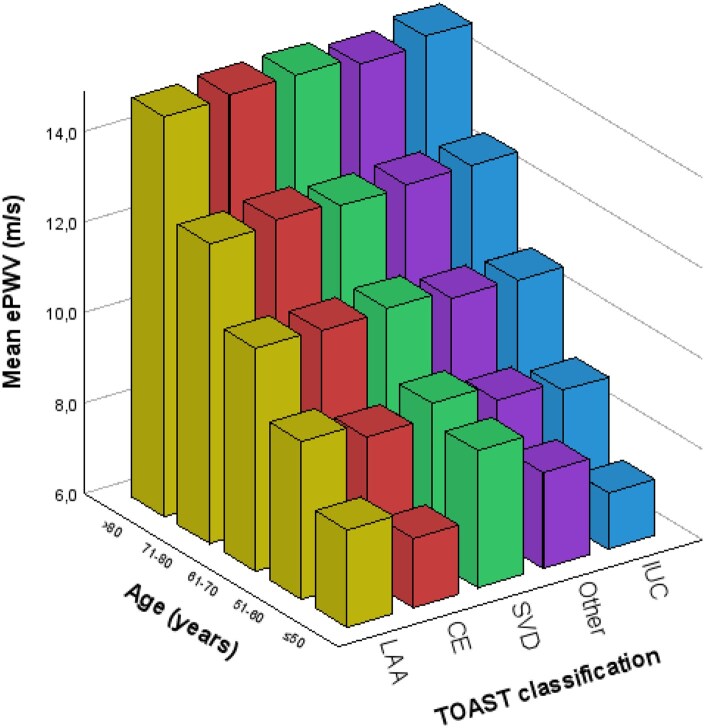
Mean ePWV across AIS subtypes by TOAST classification and age category.

When stratified by age, significant differences between TOAST categories were evident only among younger patients (≤60 years) (Figure 1). In individuals aged ≤50 years, patients with SVD had the highest mean ePWV (9.0 m/s), while those with CE and IUC had the lowest (7.5 m/s and 7.2 m/s, respectively). In the 51-60 years subgroup, elevated ePWV was again observed in patients with SVD and LAA (both 9.5 m/s), whereas patients with IUC had the lowest mean values (8.9 m/s) (Table 2, Figure 1).

Further exploratory analyses of patients ≤60 years demonstrated that those with SVD and LAA strokes were more frequently male and had a higher prevalence of hypertension, diabetes, dyslipidemia, and an atherogenic lipid profile. They also exhibited higher 24h-SBP and 24h-DBP, lower rates of pre-stroke disability, and milder stroke severity based on NIHSS score (Table S3).

Additional exploratory analyses were conducted in younger patients (≤50 years) due to the strong statistical significance and larger difference observed in ePWV values (9.1 vs. 7.5 m/s, *P* < .001) between those with SVD and all other AIS subtypes. This approach is further supported by the fact that, although a formal definition of young stroke is lacking, most studies define young ischemic stroke as occurring between 18 and 50 years of age,^23^^,^^24^ a subgroup of particular clinical and research interest due to its distinct risk profile and underlying mechanisms. In the present study, young AIS patients with SVD were approximately four years older on average, were more frequently male, and had at least a two-fold higher prevalence of hypertension, diabetes, dyslipidemia, and smoking, along with an atherogenic lipid profile. They also exhibited higher 24h-SBP and 24h-DBP (Table S4).

### Comparison of ePWV across stroke severity categories

**Figure 2:**
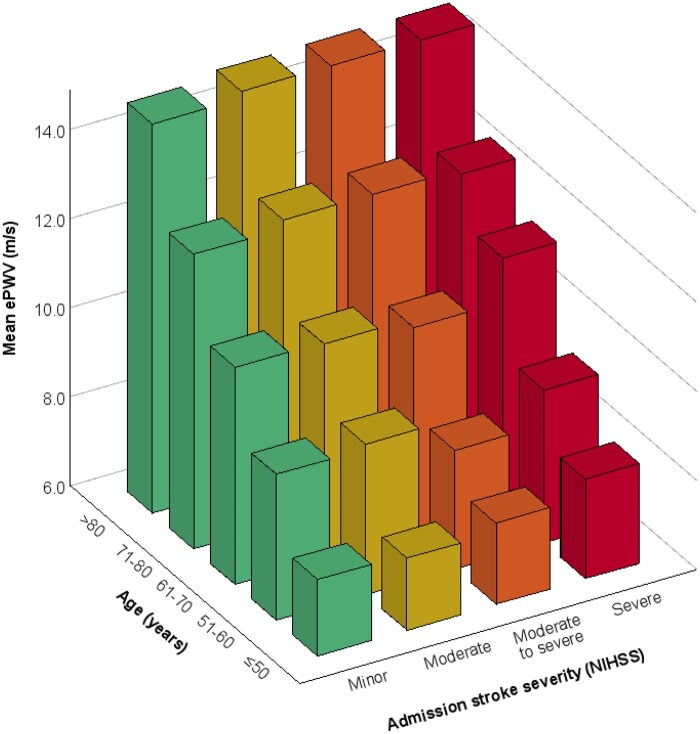
Mean ePWV by stroke severity at admission and age group.

When ePWV was examined across stroke severity strata defined by NIHSS, mean values increased progressively with stroke severity, ranging from 11.6 m/s in minor strokes to 13.5 m/s in severe strokes. Age-stratified analyses confirmed this association in patients aged 61-70 years and in those older than 80 years, where the gradient of higher ePWV with greater NIHSS scores remained statistically significant (Table 3, Figure 2).

**Table 3:** Comparison of mean ePWV by admission stroke severity AIS.

NIHSS at admission (*n* = 2711)	Minor (NIHSS 0-4)	Moderate (NIHSS 5-15)	Moderate to severe (NIHSS 16-20)	Severe (NIHSS 21-42)	*P*
**ePWV (m/s)**	11.6 ± 2.6	12.6 ± 2.7	13.1 ± 2.6	13.4 ± 2.4	<.001*
***Age category (years)***					
**≤50**	7.7 ± 1.2 (209)	7.6 ± 1.2 (65)	7.8 ± 1.1 (8)	8.2 ± 1.0 (11)	.259
**51-60**	9.3 ± 1.0 (148)	9.4 ± 1.3 (74)	8.7 ± 1.0 (13)	9.4 ± 1.0 (14)	.311
**61-70**	10.9 ± 0.9 (313)	10.8 ± 1.3 (159)	10.6 ± 0.9 (31)	11.6 ± 1.6 (30)	.016*
**71-80**	12.6 ± 1.0 (376)	12.8 ± 1.2 (269)	12.8 ± 1.2 (74)	12.7 ± 1.2 (70)	.206
**>80**	14.7 ± 1.2 (313)	15.0 ± 1.3 (340)	15.3 ± 1.2 (95)	15.0 ± 1.3 (126)	<.001*

Data are mean ± SD (number), *P*value derived from the Kruskal-Wallis Test tests and statistically significant values (*P* < .05) have been indicated bold with an asterisk (*).

Abbreviations: AIS: acute ischemic stroke, ePWV: estimated pulse wave velocity, NIHSS: National Institutes of Health stroke scale.

### Predictive value

Multivariable logistic regression analyses were performed to evaluate the independent predictive value of ePWV, treated as a continuous variable, for poor functional outcome (mRS >2) at discharge and at 3 months (Table 4). Higher ePWV was associated with increased odds of poor outcome at both time points: discharge (adjusted odds ratio [aOR]: 1.26, 95% confidence interval [CI]: 1.12-1.42, *P* < .001) and 90 days (aOR 1.24, 95% CI 1.05-1.45, *P* = .009), per 1 m/s increase in ePWV. These associations remained significant after adjustment for age, sex, BMI (included only in the 90-day model), total cholesterol, LDL cholesterol (included only at discharge model), triglycerides (included only at discharge model), eGFR, 24-hour heart rate, admission NIHSS score, and the presence of hypertension, atrial fibrillation, CAD, prior stroke or TIA, and smoking status (Table 4).

**Table 4:** Analyses of ePWV as an independent predictor of poor outcome at discharge and 90 days.

Outcome	AIS (n)	Univariate		AIS (n)	Multivariate	
		OR (95% CI)	*P*		aOR (95% CI)	*P*
**mRS > 2 at discharge^1^**	2307	1.39 (1.34-1.45)	<.001*	1913	1.26 (1.12-1.42)	<.001*
**mRS > 2 at 90 days^2^**	1550	1.56 (1.47-1.64)	<.001*	1225	1.24 (1.05-1.45)	.009*

Multivariate logistic regression analysis included all statistically significant (*P* < .1) clinical/laboratory variables from initial univariate analysis:

1 Age, Sex, Hypertension, Atrial fibrillation, Coronary artery disease, Previous stroke or TIA, Smoking, NIHSS, 24-h HR, Total cholesterol, Triglycerides, LDL cholesterol, eGFR.

2 Age, Sex, BMI, Hypertension, Diabetes, Atrial fibrillation, Coronary artery disease, Previous stroke or TIA, Smoking, NIHSS, 24-h HR, Total cholesterol, eGFR. Statistically significant values (*P* < .05) have been indicated bold with an asterisk (*). AIS: acute ischemic stroke, aOR: adjusted odds ratio, ePWV: estimated pulse wave velocity, mRS: modified Rankin Scale, CI: confidence interval. Odds ratios are expressed per 1 m/s increase in ePWV.

In sensitivity analyses (Tables S5 and S6), ePWV remained significantly associated with poor outcome at discharge and at 90 days after adjustment for age and 24h-SBP or 24h-PP, whereas results were attenuated in models including both 24h-SBP and 24h-DBP or only 24h-DBP at 90 days. In contrast, 24h-PP showed limited and inconsistent associations with outcomes (Table S7), remaining significant only in selected models and not in those including ePWV. Multicollinearity analyses (Table S8) demonstrated severe collinearity when ePWV was modeled with age and 24h-SBP or 24h-DBP (VIFs >15), whereas models including 24h-PP showed moderate collinearity (VIFs 7-8). In residualized analyses (Table S9), ePWV independent of age and 24h-PP remained significantly associated with poor outcomes both at discharge (aOR 1.21, 95% CI 1.05-1.40, *P* = .010) and at 90 days (aOR 1.34, 95% CI 1.11-1.62, *P* = .002).

In additional analyses stratified by age groups (Table S10), the association between ePWV and poor functional outcome was directionally consistent across strata, reaching statistical significance primarily in patients aged 71-80 years.

## Discussion

This study is the first large-scale, multinational pooled analysis to investigate ePWV in patients with acute ischemic stroke. By utilizing individual patient data from diverse cohorts, we demonstrated that ePWV, calculated from 24h-BPM performed soon after stroke onset, was associated with functional outcomes at discharge and at 90 days. These findings highlight ePWV as a readily available proxy of cfPWV and vascular aging in the context of AIS. Of particular relevance, the markedly higher ePWV observed in the youngest patients (≤50 years) with SVD compared with all other AIS subtypes highlights an accelerated vascular aging phenotype that emerges even early in life.

From the comparison of general patient characteristics, individuals with higher ePWV were predominantly older, more frequently female, and had a higher prevalence of classical cardiovascular risk factors, including hypertension, atrial fibrillation, coronary artery disease, and previous cerebrovascular events. They also presented with more severe strokes and were more likely to have cardioembolic etiology. Conversely, patients with lower ePWV were typically younger males with fewer conventional comorbidities but unexpectedly showed higher smoking rates (the “smoking paradox”)^25^^,^^26^ and a more atherogenic lipid profile. As ePWV is understood to reflect early arterial stiffening driven primarily by hemodynamic pressure load and vascular remodeling,^20^ these findings align with the notion that, in such patients, stroke risk may be influenced less by vascular aging (arteriosclerosis) and more by accelerated atherosclerotic processes.

Interestingly, in patients under 50 years, ePWV was highest among those with SVD, who also displayed a disproportionate burden of cardiovascular risk factors, including higher rates of hypertension, diabetes, dyslipidemia, smoking, and an atherogenic lipid profile, despite their younger age. These findings suggest that, in this very young AIS subgroup, elevated ePWV may serve as an early marker of underlying arteriosclerotic or pre-atherosclerotic vascular burden.

Our study also showed that ePWV was associated with poor functional outcome, measured by the modified Rankin Scale, both at hospital discharge and at 90 days. Importantly, this predictive value was confirmed even after adjusting for established outcome predictors, including stroke severity, comorbidities, and biochemical markers. However, given that ePWV is mathematically derived from age and blood pressure, additional analyses were performed to account for potential confounding and shared variance. Sensitivity analyses showed that the association remained statistically significant in most models, particularly when pulse pressure was used, whereas models including diastolic blood pressure showed some attenuation. In age-stratified analyses, the association between ePWV and poor functional outcome was directionally consistent across age groups but reached statistical significance mainly in patients aged 71-80 years, likely reflecting greater statistical power and the more pronounced impact of arterial stiffness in older individuals, while the absence of statistical significance in younger groups may also relate to the smaller sample sizes within these strata. The simplicity of ePWV, requiring only age and blood pressure data, makes it highly applicable in clinical practice without the need for specialized equipment or training.

The strong predictive ability of ePWV likely reflects the combined contribution of its components: age and blood pressure. Sensitivity analyses suggested that much of the prognostic effect was mediated by these two variables. Age is a well-established predictor of functional recovery after stroke,^27^^,^^28^ while elevated blood pressure in the acute phase, often described as acute hypertensive response, has consistently been linked to worse outcomes.^29^ Continuous 24h-BPM provides a more reliable assessment of hemodynamic status than single blood pressure recordings, as it reduces measurement variability, observer bias, and admission-related stress responses.^30^ Previous studies have shown that 24-hour blood pressure monitoring correlates more strongly with target organ damage and cardiovascular events than office measurements, supporting its role in improving prognostic assessment in AIS.^30^^,^^31^

Our results are consistent with previous research showing that ePWV predicts cardiovascular morbidity and mortality independent of traditional risk factors.^13^ Moreover, prior analyses in AIS populations have compared ePWV with other indirect vascular aging indices, such as the early vascular aging ambulatory score (EVAAs)^14^ and pulse pressure (PP), and consistently found ePWV to be superior.^15^ In the present study, 24h-PP showed limited and inconsistent associations with outcomes and did not retain significance in models including ePWV, suggesting that ePWV may capture additional aspects of vascular aging beyond conventional blood pressure-derived metrics. Thus, incorporating ePWV into stroke risk stratification or by guiding and selecting those stroke patients that merit cfPWV measurement, could refine prognostic models and improve patient counseling.

Multicollinearity analyses demonstrated substantial collinearity when ePWV was modeled together with age and 24h-SBP or 24h-DBP, whereas models including 24h-PP showed moderate and acceptable levels of collinearity. To further address this issue, residualized analyses were performed, demonstrating that ePWV remained significantly associated with outcomes even after accounting for age and 24h-PP. Nevertheless, these findings indicate that the predictive value of ePWV partly reflects shared variance with its constituent variables and should therefore be interpreted with caution.

From a clinical perspective, assessing vascular aging in hospitalized AIS patients could enhance risk communication, support secondary prevention strategies, and potentially improve treatment adherence.^32^^,^^33^ It also raises the possibility that therapies targeting vascular aging and arterial stiffness may improve long-term outcomes in selected patients.^11^^,^^34^ Particularly in younger AIS patients with a seemingly low conventional cardiovascular burden, ePWV may provide critical information for identifying hidden vascular risk and guiding more personalized interventions.

Several limitations should be acknowledged. First, the observational design, with two cohorts being retrospective, introduces potential biases and limits causal inference. Second, although the pooled data increased statistical power and generalizability, the heterogeneity of study protocols and populations may have introduced residual confounding. In particular, variability in the timing of 24-hour blood pressure monitoring (hyperacute, acute, and subacute phases) across cohorts may have influenced autonomic status, hemodynamic measurements, and thus ePWV estimates, potentially affecting outcome associations. Third, no corrections for multiple testing were applied, raising the possibility of type I error, thus, the findings should be interpreted as exploratory. Furthermore, data on acute stroke treatments, antihypertensive therapy, and blood pressure-lowering strategies during monitoring were limited and not consistently available, precluding adjustment for potential treatment-related confounding. Finally, while ePWV provides practical advantages, its reliance on age and blood pressure measurements may limit its ability to capture the full complexity of arterial stiffness compared with direct measures such as carotid-femoral PWV.

## Conclusions

In this large, multinational analysis, ePWV derived from 24h-BPM was associated with functional outcomes in patients with AIS, both at discharge and at 90 days. While this association partly reflects shared variance with age and blood pressure parameters, additional and residualized analyses suggest that ePWV may capture aspects of vascular aging beyond conventional measures such as pulse pressure. Beyond its predictive role, ePWV offers a simple and widely accessible method for estimating vascular aging and arterial stiffness in routine clinical practice. Its utility may be particularly relevant for younger AIS patients with SVD, in whom it may help identify vascular risk not readily captured by conventional risk factor assessment.

The integration of ePWV into clinical care could improve risk stratification or guide cfPWV diagnostic measurement, enhance secondary prevention strategies, and potentially inform novel therapeutic approaches aimed at vascular aging.

## Supplementary Material

hpag066_Supplementary_Data

## Data Availability

Unpublished data related to this article can be shared upon reasonable request to the corresponding author. However, individual participant data from separate studies and registries cannot be shared, as the license was granted exclusively for this study. Requests for such data should be directed to the specific clinical research centers involved.
