# Supplementary material for: Estimated pulse wave velocity predicts outcomes after acute ischemic stroke: a multinational cohort analysis
Source: Am J Hypertens. 2026 Jun 25;39(9):1142–50. doi: 10.1093/ajh/hpag066 (PMC13473874; doi:10.1093/ajh/hpag066)
Supplement: Supplementary file 1 [file hpag066_supplementary_data.zip › Supplemental_ePWV_AIS_R2.docx]

**SUPPLEMENTAL**

**Estimated Pulse Wave Velocity Predicts Outcomes After Acute Ischemic Stroke: A Multinational Cohort Analysis**

| **Table S1.** **Baseline characteristics of AIS patients included in the pooled IPD analysis.** | | | | | | | | |
| --- | --- | --- | --- | --- | --- | --- | --- | --- |
| **Study/Registry** | **Institute/Town/Country** | **Design** | **AIS** | **Study Period** | **Stroke onset to ABPM** | **ABPM device** | **Intervals of**  **24h-ABPM** | **Outcome** |
| ASTRAL Registry (1) | Lausanne University Hospital (CHUV), Lausanne, Switzerland | Prospective Cohort | 629 | 2023 | 51.2 hrs | NR | NR | mRS (discharge) |
| Local Registry | “Laiko” General Hospital, Athens, Greece | Prospective Cohort | 65 | 10/2020-2/2021 | 47.4 hrs | Mobil-O-Graph 24h PWA v.20 (I.E.M. GmbH, Germany) | 15-min (day) & 30-min (night) | mRS (discharge, 90 days), Stroke recurrence |
| Published Study (2) | Siena University Hospital, Siena, Italy | Prospective Cohort | 148 | NR | 24 hrs | Bedside Monitor Life Scope I BSM-2303K (International Div., Nihon Kohden Corp., Tokyo, Japan) | 30-min (06:00-00:00) & 60-min (00:00-06:00) | mRS (discharge, 90 days) |
| SECRETO study (3) | University Hospitals of Helsinki (HUS), Kuopio, Tampere and Turku, Finland | Prospective Multicentre Case-control | 132 | 10/2015-2/2020 | 20.4 days | Spacelabs Healthcare (Snoqualmie, WA, USA) or Novacor (Rueil-Malmaison, France) or Schiller AG (Baar, Switzerland) | 20-min (06:00-22:00) & 30-min (22:00-06:00) | mRS (90 days) |
| Published Study (4) | University Hospital Zurich, Zurich, Switzerland | Cross-sectional | 105 | NR | 70 days | Mobil-O-Graph 24h PWA v.20 (I.E.M. GmbH, Germany) | 15-min (08:00-22:00) & 30-min (22:00-08:00) | NIHSS |
| Published Study (5) | Hiroshima University Hospital, Chikamori Hospital, Hiroshima and Kochi, Japan | Retrospective Double-centre Cohort | 856 | 4/2010-3/2018 | 7.8 days | FB-270 (Fukuda Denshi Company Ltd, Tokyo, Japan) | 30-min | mRS (discharge, 90 days) |
| PREVISE Study (6) | University Hospitals of Thessaloniki “AHEPA”, Larissa and Ioannina, Greece | Prospective Multicentre Cohort | 228 | 11/2013-9/2017 | 18.9 hrs | TM-2430 (A&D Company Ltd, Tokyo, Japan) | 20-min | mRS (discharge, 90 days, 1 year) |
| Published Study (7) | Hanyang University Hospital, Seoul, Republic of Korea | Retrospective Cohort | 369 | 3/2011-2/2014 | 7-14 days | TM-2430 (A&D Medical, Milpitas, CA, USA) | 15-min (day) & 30-min (night) | mRS (discharge), Stroke recurrence |
| Athens Stroke Registry (8) | “Alexandra” General Hospital, Athens, Greece | Prospective Cohort | 239 | 2006-2010 | 10.8 hrs | SpaceLabs 90207 (Spacelabs Inc, Redmond, WA, USA) | 15-min | mRS (discharge, 90 days, 5 years), Stroke recurrence |
| PhD Thesis (9) | “Papageorgiou” General Hospital, Thessaloniki, Greece | Prospective Cohort | 84 | 9/2007-7/2009 | 24.6 hrs | SpaceLabs 90207 (Spacelabs Inc, Redmond, WA, USA) | NR | Death (1 year) |
| GTN-2 Trial (10) | Nottingham City Hospital, Nottingham, United Kingdom | Randomized Controlled Trial | 84* | 12/1998-6/2001 | 55.1 hours | SpaceLabs 90207 (Spacelabs Inc, Redmond, WA, USA) | 20-min (07:00-23:00) & 60-min (23:00-07:00) | mRS (90 days) |
| The "Stroke onset to ABPM" column displays the mean time in hours or days. ABPM: ambulatory blood pressure monitoring, AIS: acute ischemic stroke (number of patients), ASTRAL: Acute STroke Registry and Analysis of Lausanne, GTN: Glyceryl trinitrate, hrs: hours, IPD: individual patient data, mRS: modified Rankin Scale, NIHSS: National Institutes of Health Stroke Scale, NR: not reported, PREVISE: Blood Pressure Variability in Acute Ischemic Stroke, SECRETO: Searching for Explanations for Cryptogenic Stroke in the Young: Revealing the Etiology, Triggers, and Outcome.  ** Included only patients with Lacunar Syndrome (LACS) as defined by the OCSP classification from the initial 90 patients.* | | | | | | | | |

| **Table S2. Characteristics of AIS patients of the studies and registries participating in the IPD pooled analysis.** | | | | | | | | | | | | |
| --- | --- | --- | --- | --- | --- | --- | --- | --- | --- | --- | --- | --- |
| **Study/Registry** | **Age (years)** | **Sex (male)** | **BMI (kg/m^2^)** | **Admission NIHSS** | **TOAST (LAA/CE/SVD/Other/IUC)** | **HT** | **DM** | **DLP** | **AF** | **CAD** | **Stroke /TIA** | **Smoking** |
| **ASTRAL Registry**  (*Lausanne, Switzerland*) (1) | 72.8±14.8 | 57.9% | 25.9±4.9 | 8 (4-16) | 21.1/35.1/5.4/11.3/27 % | 72.5% | 19.2% | 68.8% | 34.5% | 18.9% | 24.4% | 22.3% |
| **Local Registry**  (*Laiko, Athens, Greece*) | 78.1±13.1 | 50.8% | 26.8±4.4 | 6 (2-14) | 15/33.3/11.7/1.7/38.3 % | 76.9% | 30.8% | 61.5% | 21.5% | 23.1% | 32.3% | 46.7% |
| **Published Study**  (*Siena, Italy*) (2) | 72.3±13.0 | 54.7% | 25.8±3.7 | 9 (5-18) | 32.4/0/0/0/67.6 % | 71.6% | 20.9% | 52.7% | 0% | 9.5% | 13.5% | 23% |
| **SECRETO study**  (Helsinki, Finland) (3) | 39.8±8.1 | 56.1% | 26.9±4.9 | 1 (0-3) | 0/0/0/0/100 % | 35.6% | 3.8% | 6.1% | 0% | 0.8% | NR | 29% |
| **Published Study**  (*Zurich, Switzerland*) (4) | 71.2±12.8 | 64.8% | 26.7±5.1 | NR | 31.4/1/20/36.2/11.4 % | 57.1% | 24.8% | 71.4% | 19% | 22.9% | 16.2% | NR |
| **Published Study**  (*Hiroshima, Kochi, Japan*) (5) | 76.5±11.8 | 55.4% | 23.1±3.8 | 3 (2-9) | 12.1/24.6/18/16.3/29.1 % | 70.8% | 31.5% | 43.2% | 23.5% | 38.6% | 11.8% | 19.5% |
| **PREVISE Study** (*Thessaloniki, Larissa, Ioannina, Greece*) (6) | 80.1±7.1 | 45.6% | 27.3±4.3 | 7 (3-16) | 16.2/35.5/7.9/1.3/39 % | 83.8% | 31.1% | 40.8% | 39% | 19.7% | 37.7% | 25.9% |
| **Published Study**  (*Hanyang, Korea*) (7) | 66.1±13.0 | 62.6% | 26.5±4.3 | 2 (1-4) | 30.6/19/33.3/4.1/13 % | 62.6% | 43.4% | 22.5% | 18.7% | 7.9% | 13.8% | 36.6% |
| **Athens Stroke Registry** (Alexandra, Athens, GR) (8) | 69.3±10.2 | 64% | 26.7±3.8 | 5 (2-16) | 22.6/32.2/26.8/2.1/16.3% | 74.5% | 31.4% | 32.2% | 31% | 20.5% | 10.9% | 39.7% |
| **PhD Thesis** (“*Papageorgiou”, Thessaloniki, Greece*) (9) | 77.4±5.9 | 48.8% | 28.9±2.4 | 0 (0-2) | NR | 82.7% | 25.9% | 29.6% | NR | NR | 34.9% | 11.1% |
| **GTN-2 Trial**  (Nottingham, UK) (10) | 72.1±11.7 | 44% | 25.0±5.4 | 11 (7.3-15) | 0/0/100/0/0 % | 41.7% | 14.3% | 4.8% | 20.2% | NR | 16.7% | 19% |
| Data are numbers (%) for categorical variables, mean ± SD for continuous variables, except NIHSS which is median (IQR). AF: atrial fibrillation, ASTRAL: Acute STroke Registry and Analysis of Lausanne, BMI: body mass index, CAD: coronary artery disease, CE: cardioembolic, DLP: dyslipidemia, DM: diabetes mellitus, GTN: Glyceryl trinitrate, HT: hypertension, IPD: individual patient data IUC: infarct of undetermined cause, LAA: large artery atherosclerotic,, NIHSS: National Institutes of Health Stroke Scale, NR: not reported, Other: stroke of other determined causes, PREVISE: Blood Pressure Variability in Acute Ischemic Stroke, SECRETO: Searching for Explanations for Cryptogenic Stroke in the Young: Revealing the Etiology, Triggers, and Outcome, SVD: small vessel disease or lacunar stroke, TIA: transient ischemic attack. | | | | | | | | | | | | |

| **Table S3. Comparison of clinical and laboratory features among AIS patients ≤60 years across TOAST subtypes.** | | | | | | | |
| --- | --- | --- | --- | --- | --- | --- | --- |
| **Variable** | **All (≤60 years)** | **LAA (n=83)** | **CE (n=51)** | **SVD (n=107)** | **Other (n=75)** | **IUC (n=243)** | **p** |
| ePWV (m/s) | 8.4±1.4 | 9.0±1.2 | 8.5±1.5 | 9.3±1.1 | 8.7±1.3 | 7.7±1.2 | **<0.001*** |
| **Demographic/somatometric data** | | | | | | | |
| Age (years) | 48.3±9.3 | 52.4±5.5 | 50.5±7.9 | 52.1±6.4 | 48.7±10.1 | 44.7±10.0 | **<0.001*** |
| Sex (male) | 376 (67.1%) | 62 (74.7%) | 27 (52.9%) | 83 (77.6%) | 57 (76.0%) | 147(60.5%) | **<0.001*** |
| BMI (kg/m^2^) | 26.1±4.8 | 26.4±4.9 | 24.9±5.3 | 25.8±4.3 | 25.5±4.3 | 26.6±4.9 | **0.045*** |
| **Clinical risk factors** | | | | | | | |
| Hypertension | 261 (46.6%) | 46 (55.4%) | 19 (37.3%) | 66 (61.7%) | 37 (48.7%) | 93 (38.3%) | **<0.001*** |
| Diabetes | 108 (19.3%) | 33 (39.8%) | 10 (19.6%) | 30 (28.0%) | 9 (12.0%) | 26 (10.7%) | **<0.001*** |
| Dyslipidemia | 209 (37.3%) | 50 (60.2%) | 20 (39.2%) | 41 (38.3%) | 30 (40.0%) | 68 (28.0%) | **<0.001*** |
| Atrial fibrillation | 36 (6.4%) | 3 (3.6%) | 24 (47.1%) | 2 (1.9%) | 2 (2.7%) | 5 (2.1%) | **<0.001*** |
| CAD | 44 (8.0%) | 11 (13.3%) | 4 (7.8%) | 7 (7.1%) | 8 (10.7%) | 14 (5.8%) | 0.232 |
| Prior stroke/TIA | 57 (13.3%) | 15 (18.3%) | 6 (11.8%) | 11 (10.3%) | 11 (14.7%) | 14 (12.6%) | 0.579 |
| Smoking | 236 (44.0%) | 43 (56.6%) | 22 (44.0%) | 58 (57.4%) | 29 (43.3%) | 84 (34.9%) | **<0.001*** |
| **24h-BPM data** | | | | | | | |
| 24-h SBP (mmHg) | 131.4±19.9 | 136.6±18.0 | 132.6±21.8 | 140.4±20.5 | 136.4 ±20.1 | 123.9 ±17.1 | **<0.001*** |
| 24-h DBP (mmHg) | 80.1±12.0 | 82.8±12.2 | 78.4±13.2 | 87.1±11.6 | 82.1±12.5 | 78.5±9.9 | **<0.001*** |
| 24-h HR (bpm) | 72.2±11.8 | 72.7±13.1 | 74.8±14.6 | 69.0±10.3 | 75.3±13.9 | 72.0±10.3 | **0.011*** |
| **Laboratory data** | | | | | | | |
| TCL (mmol/l) | 5.0±1.6 | 5.6±2.6 | 4.6±1.1 | 5.3±1.5 | 4.9±1.5 | 4.8±1.1 | **<0.001*** |
| TRG (mmol/l) | 1.7±1.6 | 2.0±1.9 | 1.7±1.6 | 1.9±1.5 | 2.0±2.2 | 1.4±0.8 | **0.005*** |
| LDL (mmol/l) | 3.1±1.2 | 3.5±2.0 | 2.7±1.0 | 3.4±1.2 | 2.9±1.2 | 3.0±1.0 | **0.002*** |
| HDL (mmol/l) | 1.2±0.4 | 1.2±0.4 | 1.3±0.4 | 1.1±0.3 | 1.3±0.5 | 1.3±0.4 | **0.009*** |
| eGFR | 93.5±22.3 | 87.7±19.1 | 84.5±25.9 | 94.1±23.4 | 89.7±26.2 | 98.3±19.6 | **<0.001*** |
| **Stroke data** | | | | | | | |
| Pre-stroke mRS>2 | 7 (2.2%) | 1 (1.6%) | 3 (6.4%) | 1 (1.3%) | 2 (3.2%) | 0 (0.0%) | 0.177 |
| Admission NIHSS | 2 (1-7) | 3 (1-7) | 5 (2-15) | 2 (1-4) | 4 (2-13) | 2 (1-6) | **<0.001*** |
| **Acute interventions** | | | | | | | |
| Thrombolysis | 131 (26.6%) | 17 (26.2%) | 21 (44.7%) | 3 (3.8%) | 13 (20.3%) | 77 (32.5%) | **<0.001*** |
| Thrombectomy | 15 (8.3%) | 2 (50.0%) | 0 (0.0%) | 0 (0.0%) | 0 (0.0%) | 13 (8.0%) | **0.034*** |
| **Outcomes** | | | | | | | |
| Discharge mRS>2 | 82 (22.9%) | 19 (26.0%) | 8 (16.0%) | 10 (11.0%) | 26 (40.0%) | 19 (24.4%) | **<0.001*** |
| 90 days mRS>2 | 21 (7.4%) | 5 (21.7%) | 0 (0.0%) | 4 (8.9%) | 3 (15.8%) | 9 (4.8%) | **0.019*** |
| Data are numbers (%) for categorical variables, mean±SD for continuous variables, except NIHSS which is median (IQR), p-values derived from the chi-squared tests and the Kruskal-Wallis tests and statistically significant values (p<0.05) have been indicated bold with an asterisk (*). 24h-BPM: 24-hour blood pressure monitoring, AIS: acute ischemic stroke, BMI: body mass index, CAD: coronary artery disease CE: cardioembolic, DBP: diastolic blood pressure, eGFR: estimated glomerular filtration rate (ml/min/1.73m2), ePWV: estimated pulse wave velocity, HDL: high-density lipoprotein cholesterol, HR: heart rate, IUC: infarct of undetermined cause, LAA: large artery atherosclerosis, LDL: low-density lipoprotein cholesterol, mRS: modified Rankin Scale, NIHSS: National Institutes of Health stroke scale, Other: stroke of other determined causes, SBP: systolic blood pressure, SVD: small vessel disease or lacunar stroke, TCL: Total cholesterol, TIA: transient ischemic attack, TOAST: Trial of Org 10172 in Acute Stroke Treatment, TRG: triglycerides. | | | | | | | |

| **Table S4. Comparison of clinical and laboratory features between AIS patients ≤50 years with SVD vs. all other TOAST subtypes.** | | | | | |
| --- | --- | --- | --- | --- | --- |
| **Variable** | **Valid values** | **AIS patients**  **≤50 years old** | **SVD**  **(n=39)** | **All other TOAST**  **(n=242)** | **p** |
| ePWV (m/s) | 281 | 7.7 ± 1.2 | 9.1 ± 1.2 | 7.5 ± 1.1 | **<0.001*** |
| **Demographic/somatometric data** | | | | | |
| Age (years) | 281 | 41.7 ± 7.4 | 45.5 ± 3.8 | 41.1 ± 7.8 | **<0.001*** |
| Sex (male) | 281 | 179 (63.7%) | 33 (84.6%) | 146 (60.3%) | **0.003*** |
| BMI (kg/m^2^) | 281 | 26.7 ± 5.0 | 26.4 ± 4.1 | 26.7 ± 5.2 | 0.938 |
| **Clinical risk factors** | | | | | |
| Hypertension | 281 | 106 (37.7%) | 25 (64.1%) | 81 (33.5%) | **<0.001*** |
| Diabetes | 281 | 34 (12.1%) | 12 (30.8%) | 22 (9.1%) | **<0.001*** |
| Dyslipidemia | 281 | 69 (24.6%) | 18 (46.2%) | 51 (21.1%) | **<0.001*** |
| Atrial fibrillation | 280 | 7 (2.5%) | 0 (0.0%) | 7 (2.9%) | 0.346 |
| CAD | 277 | 9 (3.2%) | 3 (8.1%) | 6 (2.5%) | 0.073 |
| Prior stroke/TIA | 149 | 18 (12.1%) | 3 (7.7%) | 15 (13.6%) | 0.328 |
| Smoking | 272 | 117 (43.0%) | 25 (69.4%) | 92 (39.0%) | **<0.001*** |
| **24h-BPM data** | | | | | |
| 24-h SBP (mmHg) | 281 | 129.2 ± 20.0 | 148.4 ± 21.6 | 126.1 ± 17.9 | **<0.001*** |
| 24-h DBP (mmHg) | 281 | 80.0 ± 12.6 | 94.0 ± 11.2 | 77.8 ± 11.3 | **<0.001*** |
| 24-h HR (bpm) | 281 | 73.0 ± 11.0 | 72.8 ± 10.1 | 73.1 ± 11.2 | 0.841 |
| **Laboratory data** | | | | | |
| TCL (mmol/l) | 269 | 4.9 ± 1.2 | 5.4 ± 1.5 | 4.8 ± 1.1 | **0.007*** |
| TRG (mmol/l) | 257 | 1.5 ± 1.0 | 2.1 ± 1.3 | 1.5 ± 0.9 | **0.001*** |
| LDL (mmol/l) | 267 | 3.1 ± 1.0 | 3.5 ± 1.2 | 3.0 ± 1.0 | **0.017*** |
| HDL (mmol/l) | 256 | 1.2 ± 0.4 | 1.1 ± 0.2 | 1.3 ± 0.4 | 0.050 |
| eGFR | 281 | 98.9 ± 21.7 | 98.0 ± 27.3 | 99.0 ± 20.7 | 0.441 |
| **Stroke data** | | | | | |
| Pre-stroke mRS>2 | 119 | 1 (0.8%) | 0 (0.0%) | 1 (1.1%) | 0.773 |
| Admission NIHSS | 272 | 2 (1-5) | 2 (1-4) | 2 (1-4) | 0.894 |
| *TOAST* |  |  |  |  |  |
| LAA | 279 | 27 (9.7%) | - | 27 (11.3%) | **<0.001*** |
| CE |  | 15 (5.4%) | - | 15 (6.3%) |  |
| SVD |  | 39 (14.0%) | 39 (100%) | - |  |
| Other |  | 33 (11.8%) | - | 33 (13.8%) |  |
| IUC |  | 165 (59.1%) | - | 165 (68.8%) |  |
| **Acute interventions** | | | | | |
| Thrombolysis | 261 | 58 (22.2%) | 1 (3.6%) | 57 (24.5%) | **0.012*** |
| Thrombectomy | 146 | 8 (5.5%) | 0 (0.0%) | 8 (5.6%) | 0.843 |
| **Outcomes** | | | | | |
| Discharge mRS>2 | 126 | 27 (21.4%) | 3 (9.1%) | 24 (25.8%) | **0.044*** |
| 90 days mRS>2 | 180 | 7 (3.9%) | 1 (5.9%) | 6 (3.7%) | 0.507 |
| Data are numbers (%) for categorical variables, mean ± SD for continuous variables, except NIHSS which is median (IQR), p-values derived from the chi-squared tests and the Mann-Whitney tests and statistically significant values (p<0.05) have been indicated bold with an asterisk (*). 24h-BPM: 24-hour blood pressure monitoring, AIS: acute ischemic stroke, BMI: body mass index, CAD: coronary artery disease CE: cardioembolic, DBP: diastolic blood pressure, eGFR: estimated glomerular filtration rate (ml/min/1.73m^2^), ePWV: estimated pulse wave velocity, HDL: high-density lipoprotein cholesterol, HR: heart rate, IUC: infarct of undetermined cause, LAA: large artery atherosclerosis, LDL: low-density lipoprotein cholesterol, NIHSS: National Institutes of Health stroke scale, Other: stroke of other determined causes, SBP: systolic blood pressure, SVD: small vessel disease or lacunar stroke, TCL: Total cholesterol, TIA: transient ischemic attack, TOAST: Trial of Org 10172 in Acute Stroke Treatment, TRG: triglycerides | | | | | |

| **Table S5. Sensitivity analyses of ePWV as an independent predictor of poor outcome at discharge and 90 days.** | | | | | | |
| --- | --- | --- | --- | --- | --- | --- |
| **Outcome** | **AIS (n)** | **Univariate** | | **AIS (n)** | **Multivariate** | |
|  |  | **OR (95% CI)** | **p** |  | **aOR (95% CI)** | **p** |
| **mRS>2 at discharge** ^1^ | 2,307 | 1.39 (1.34-1.45) | **<0.001*** | 1,913 | 1.30 (1.03-1.64) | **0.026*** |
| **mRS>2 at 90 days** ^2^ | 1,550 | 1.56 (1.47-1.64) | **<0.001*** | 1,225 | 1.35 (0.85-2.13) | 0.202 |
| Multivariate logistic regression analysis included all statistically significant (p<0.1) clinical/laboratory variables from initial univariate analysis: ^1^ Age, Sex, Hypertension, Atrial fibrillation, Coronary artery disease, Previous stroke or TIA, Smoking, NIHSS, 24-h SBP, 24-h HR, Total cholesterol, Triglycerides, LDL cholesterol, eGFR, ^2^ Age, Sex, BMI, Hypertension, Diabetes, Atrial fibrillation, Coronary artery disease, Previous stroke or TIA, Smoking, NIHSS, 24-h SBP, 24-h DBP, 24-h HR, Total cholesterol, eGFR. Statistically significant values (p <0.05) have been indicated bold with an asterisk (*). AIS: acute ischemic stroke, aOR: adjusted odds ratio, ePWV: estimated pulse wave velocity, mRS: modified Rankin Scale, CI: confidence interval. All odds ratios for ePWV are expressed per 1 m/s increase. | | | | | | |

| **Table S6. Sensitivity analyses of ePWV as an independent predictor of poor outcome at discharge and 90 days adjusting for age, 24-h systolic or 24-h diastolic blood pressure or 24-h pulse pressure.** | | | | | | | |
| --- | --- | --- | --- | --- | --- | --- | --- |
| **Outcome** | **AIS (n)** | **Multivariate (+ 24-h SBP)** | | **Multivariate (+ 24-h DBP)** | | **Multivariate (+ 24-h PP)** | |
|  |  | **aOR (95% CI)** | **p** | **aOR (95% CI)** | **p** | **aOR (95% CI)** | **p** |
| **mRS>2 at discharge** ^1^ | 1,913 | 1.30 (1.03-1.64) | **0.026*** | 1.55 (1.27-1.90) | **<0.001*** | 1.21 (1.05-1.40) | **0.010*** |
| **mRS>2 at 90 days** ^2^ | 1,225 | 1.50 (1.10-2.06) | **0.012*** | 1.08 (0.79-1.48) | 0.626 | 1.34 (1.11-1.62) | **0.002*** |
| Multivariate logistic regression analysis included all statistically significant (p<0.1) clinical/laboratory variables from initial univariate analysis: ^1^ Age, Sex, Hypertension, Atrial fibrillation, Coronary artery disease, Previous stroke or TIA, Smoking, NIHSS, 24-h HR, Total cholesterol, Triglycerides, LDL cholesterol, eGFR, (plus 24-h SBP or 24-h DBP or 24-h PP) ^2^ Age, Sex, BMI, Hypertension, Diabetes, Atrial fibrillation, Coronary artery disease, Previous stroke or TIA, Smoking, NIHSS, 24-h HR, Total cholesterol, eGFR, (plus 24-h SBP or 24-h DBP or 24-h PP). Statistically significant values (p <0.05) have been indicated bold with an asterisk (*). AIS: acute ischemic stroke, aOR: adjusted odds ratio, CI: confidence interval, DBP: diastolic blood pressure, ePWV: estimated pulse wave velocity, mRS: modified Rankin Scale, PP: pulse pressure, SBP: systolic blood pressure. All odds ratios for ePWV are expressed per 1 m/s increase. | | | | | | | |

| **Table S7. Sensitivity analyses of 24-h pulse pressure as an independent predictor of poor outcome at discharge and 90 days adjusting for age, 24-h systolic or 24h-diastolic blood pressure or ePWV.** | | | | | | | |
| --- | --- | --- | --- | --- | --- | --- | --- |
| **Outcome** | **AIS (n)** | **Multivariate (+ 24-h SBP)** | | **Multivariate (+ 24-h DBP)** | | **Multivariate (+ ePWV)** | |
|  |  | **aOR (95% CI)** | **p** | **aOR (95% CI)** | **p** | **aOR (95% CI)** | **p** |
| **mRS>2 at discharge** ^1^ | 1,913 | 1.01 (0.99-1.02) | 0.510 | 1.01 (1.00-1.02) | **0.007*** | 1.01 (0.99-1.02) | 0.308 |
| **mRS>2 at 90 days** ^2^ | 1,225 | 0.98 (0.96-0.99) | **0.025*** | 0.99 (0.99-1.01) | 0.699 | 0.99 (0.98-1.00) | 0.125 |
| Multivariate logistic regression analysis included all statistically significant (p<0.1) clinical/laboratory variables from initial univariate analysis: ^1^ Age, Sex, Hypertension, Atrial fibrillation, Coronary artery disease, Previous stroke or TIA, Smoking, NIHSS, 24-h HR, Total cholesterol, Triglycerides, LDL cholesterol, eGFR, (plus 24-h SBP or 24-h DBP or ePWV) ^2^ Age, Sex, BMI, Hypertension, Diabetes, Atrial fibrillation, Coronary artery disease, Previous stroke or TIA, Smoking, NIHSS, 24-h HR, Total cholesterol, eGFR, (plus 24-h SBP or 24-h DBP or ePWV). Statistically significant values (p <0.05) have been indicated bold with an asterisk (*). AIS: acute ischemic stroke, aOR: adjusted odds ratio, CI: confidence interval, DBP: diastolic blood pressure, ePWV: estimated pulse wave velocity, mRS: modified Rankin Scale, PP: pulse pressure, SBP: systolic blood pressure. All odds ratios for 24h-PP are expressed per 1 mmHg increase.. | | | | | | | |

| **Table S8. Assessment of multicollinearity among predictors.** | | | | | | | |
| --- | --- | --- | --- | --- | --- | --- | --- |
| **Outcome** | **Variable** | Model A: ePWV + Age + SBP | | Model B:ePWV + Age + DBP | | Model C: ePWV + Age + PP | |
|  |  | **Tolerance** | **VIF** | **Tolerance** | **VIF** | **Tolerance** | **VIF** |
| **mRS at discharge** | ePWV | **0.05** | **18.9** | **0.06** | **16.5** | 0.13 | 7.8 |
|  | Age | **0.06** | **17.1** | **0.06** | **16.6** | 0.14 | 7.2 |
|  | 24-h SBP | 0.31 | 3.23 | - | - | - | - |
|  | 24-h DBP | - | - | 0.34 | 2.9 | - | - |
|  | 24-h PP | - | - | - | - | 0.74 | 1.3 |
| **mRS at 90 days** | ePWV | **0.06** | **17.0** | **0.07** | **15.4** | 0.12 | 8.4 |
|  | Age | **0.07** | **13.9** | **0.07** | **14.7** | 0.14 | 7.4 |
|  | 24-h SBP | 0.37 | 2.7 | - | - |  |  |
|  | 24-h DBP | - | - | 0.42 | 2.4 |  |  |
|  | 24-h PP | - | - | - | - | 0.72 | 1.4 |
| Multicollinearity was assessed using variance inflation factors (VIF) derived from linear regression models. VIF values >10 and tolerance <0.1 were considered indicative of severe multicollinearity. Values exceeding these thresholds are shown in bold. DBP: diastolic blood pressure, ePWV: estimated pulse wave velocity, mRS: modified Rankin Scale, PP: pulse pressure, SBP: systolic blood pressure, VIF: variance inflation factor. | | | | | | | |

| **Table S9. Association of residualized ePWV (adjusted for age and 24-h pulse pressure) with poor outcome at discharge and 90 days.** | | | | | | |
| --- | --- | --- | --- | --- | --- | --- |
| **Outcome** | **AIS (n)** | **Univariate** | | **AIS (n)** | **Multivariate** | |
|  |  | **OR (95% CI)** | **p** |  | **aOR (95% CI)** | **p** |
| **mRS>2 at discharge** ^1^ | 2,307 | 1.46 (1.32-1.60) | **<0.001*** | 1,913 | 1.21 (1.05-1.40) | **0.010*** |
| **mRS>2 at 90 days** ^2^ | 1,550 | 1.34 (1.20-1.49) | **<0.001*** | 1,225 | 1.34 (1.11-1.62) | **0.002*** |
| Residualized ePWV represents the residuals obtained from linear regression of ePWV on age and 24-h pulse pressure, and reflects the component of ePWV independent of these variables. Multivariate logistic regression analysis included all statistically significant (p<0.1) clinical/laboratory variables from initial univariate analysis: ^1^ Age, Sex, Hypertension, Atrial fibrillation, Coronary artery disease, Previous stroke or TIA, Smoking, NIHSS, 24-h HR, Total cholesterol, Triglycerides, LDL cholesterol, eGFR, residualized-ePWV and 24-h PP; ^2^ Age, Sex, BMI, Hypertension, Diabetes, Atrial fibrillation, Coronary artery disease, Previous stroke or TIA, Smoking, NIHSS, 24-h HR, Total cholesterol, eGFR, residualized-ePWV and 24-h PP. Statistically significant values (p <0.05) have been indicated bold with an asterisk (*). AIS: acute ischemic stroke, aOR: adjusted odds ratio, ePWV: estimated pulse wave velocity, mRS: modified Rankin Scale, CI: confidence interval. All odds ratios for residualized ePWV are expressed per 1 m/s increase. | | | | | | |

| **Table S10. Association of ePWV with poor functional outcome at discharge and 90 days across age groups (decade-stratified analysis).** | | | | | | |
| --- | --- | --- | --- | --- | --- | --- |
| **Outcome** | **AIS (n)** | **Univariate** | | **AIS (n)** | **Multivariate** | |
|  |  | **OR (95% CI)** | **p** |  | **aOR (95% CI)** | **p** |
|  |  |  |  |  |  |  |
| **mRS>2 at discharge** ^1^ | 2,307 | 1.39 (1.34-1.45) | **<0.001*** | 1,913 | 1.34 (1.27-1.43) | **<0.001*** |
|  |  |  |  |  |  |  |
| *Age category* |  |  |  |  |  |  |
| ≤ 50 years | 146 | 1.08 (0.78-1.49) | 0.654 | 118 | 1.44 (0.79-2.64) | 0.235 |
| 51-60 years | 212 | 0.94 (0.71-1.25) | 0.688 | 170 | 0.93 (0.52-1.51) | 0.771 |
| 61-70 years | 479 | 1.17 (0.98-1.40) | 0.077 | 421 | 1.31 (0.99-1.72) | 0.057 |
| 71-80 years | 675 | 1.40 (1.21-1.61) | **<0.001*** | 554 | 1.37 (1.07-1.74) | **0.011*** |
| > 80 years | 795 | 1.42 (1.25-1.61) | **<0.001*** | 650 | 1.03 (0.80-1.32) | 0.814 |
|  |  |  |  |  |  |  |
| **mRS>2 at 90 days** ^2^ | 1,550 | 1.56 (1.47-1.64) | **<0.001*** | 1,225 | 1.41 (1.31-1.53) | **<0.001*** |
|  |  |  |  |  |  |  |
| *Age category* |  |  |  |  |  |  |
| ≤ 50 years | 182 | 1.56 (0.94-2.59) | 0.086 | 45 | - | - |
| 51-60 years | 111 | 1.20 (0.77-1.85) | 0.419 | 93 | 1.02 (0.45-2.31) | 0.962 |
| 61-70 years | 286 | 1.40 (1.13-1.74) | **0.002*** | 252 | 1.40 (0.98-2.00) | 0.067 |
| 71-80 years | 463 | 1.41 (1.19-1.66) | **<0.001*** | 388 | 1.46 (1.09-1.96) | **0.011*** |
| > 80 years | 508 | 1.43 (1.21-1.69) | **<0.001*** | 447 | 1.03 (0.76-1.40) | 0.839 |
| Multivariate logistic regression analysis included all statistically significant (p<0.1) clinical/laboratory variables from initial univariate analysis: ^1^ Age, Sex, Hypertension, Atrial fibrillation, Coronary artery disease, Previous stroke or TIA, Smoking, NIHSS, 24-h HR, Total cholesterol, Triglycerides, LDL cholesterol, eGFR, ^2^ Age, Sex, BMI, Hypertension, Diabetes, Atrial fibrillation, Coronary artery disease, Previous stroke or TIA, Smoking, NIHSS, 24-h HR, Total cholesterol, eGFR. Statistically significant values (p <0.05) have been indicated bold with an asterisk (*). AIS: acute ischemic stroke, aOR: adjusted odds ratio, ePWV: estimated pulse wave velocity, mRS: modified Rankin Scale, CI: confidence interval. Odds ratios are expressed per 1 m/s increase in ePWV. | | | | | | |

**Supplemental References**

1.	Michel P, Odier C, Rutgers M, Reichhart M, Maeder P, Meuli R, et al. The Acute STroke Registry and Analysis of Lausanne (ASTRAL): design and baseline analysis of an ischemic stroke registry including acute multimodal imaging. Stroke. 2010;41(11):2491-8.

2.	Acampa M, Lazzerini PE, Manfredi C, Guideri F, Tassi R, Domenichelli C, et al. Non-stenosing Carotid Atherosclerosis and Arterial Stiffness in Embolic Stroke of Undetermined Source. Front Neurol. 2020;11:725.

3.	Tulkki L, Martinez-Majander N, Haapalahti P, Tolppanen H, Sinisalo J, Repo O, et al. 24-hour ambulatory blood pressure and cryptogenic ischemic stroke in young adults. Ann Med. 2023;55(1):2203513.

4.	Rejmer P, Palla A, Schulz EG, Neumann CL, Hund M, Hermann M. Ambulatory blood pressure monitoring on admission in survivors of recent stroke entering inpatient rehabilitation. J Hum Hypertens. 2020;34(5):364-71.

5.	Naito H, Hosomi N, Kuzume D, Nezu T, Aoki S, Morimoto Y, et al. Increased blood pressure variability during the subacute phase of ischemic stroke is associated with poor functional outcomes at 3 months. Sci Rep. 2020;10(1):811.

6.	Kakaletsis N, Ntaios G, Milionis H, Karagiannaki A, Chouvarda I, Dourliou V, et al. Prognostic significance of 24-h blood pressure and variability indices in the outcome of acute ischaemic stroke. Intern Med J. 2023;53(7):1137-46.

7.	Kwon HS, Kim JY, Choi H, Lee SJ, Koh SH, Lee YJ, et al. Association between nocturnal blood pressure variation and wake-up ischemic stroke. J Clin Neurosci. 2017;44:210-3.

8.	Zis P, Vemmos K, Spengos K, Manios E, Zis V, Dimopoulos MA, et al. Ambulatory blood pressure monitoring in acute stroke: pathophysiology of the time rate of blood pressure variation and association with the 1-year outcome. Blood Press Monit. 2013;18(2):94-100.

9.	Gamvrili L. Circadian blood pressure variation after acute ischemic stroke: Relationship with other hemodynamic parameters. National Documentation Centre: Aristotle University of Thessaloniki, Thessaloniki, Greece; 2011.

10.	Rashid P, Weaver C, Leonardi-Bee J, Bath F, Fletcher S, Bath P. The effects of transdermal glyceryl trinitrate, a nitric oxide donor, on blood pressure, cerebral and cardiac hemodynamics, and plasma nitric oxide levels in acute stroke. J Stroke Cerebrovasc Dis. 2003;12(3):143-51.
